# High-Power-Density Miniaturized VLF Antenna with Nanocrystalline Core for Enhanced Field Strength

**DOI:** 10.3390/nano15141062

**Published:** 2025-07-09

**Authors:** Wencheng Ai, Huaning Wu, Lin Zhao, Hui Xie

**Affiliations:** School of Electrical Engineering, Naval University of Engineering, Wuhan 430030, China; m24385404@nue.edu.cn (W.A.); 0909031064@nue.edu.cn (L.Z.)

**Keywords:** very low frequency, nanocrystalline alloys, high power capacity, enhanced radiation

## Abstract

In order to break through the difficulties with a very-low-frequency (VLF) miniaturized antenna with small power capacity and low radiation efficiency, this paper proposes a high-radiation-field-strength magnetic loop antenna based on a nanocrystalline alloy magnetic core. A high-permeability nanocrystalline toroidal core (*μ_r_* = 50,000, *B_s_* = 1.2 T) is used to optimize the thickness-to-diameter ratio (t = 0.08) and increase the effective permeability to 11,000. The Leeds wires, characterized by their substantial carrying capacity, are manufactured through a toroidal winding process. This method results in a 68% reduction in leakage compared to traditional radial winding techniques and enhances magnetic induction strength by a factor of 1.5. Additionally, this approach effectively minimizes losses, thereby facilitating support for kilowatt-level power inputs. A cascaded LC resonant network (resonant capacitance 2.3 μF) and ferrite balun transformer (power capacity 3.37 kW) realize a 20-times amplification of the input current. A series connection of a high-voltage isolation capacitor blocks DC bias noise, guaranteeing the stable transmission of 1200 W power, which is 6 times higher than the power capacity of traditional ring antenna. At 7.8 kHz frequency, the magnetic field strength at 120 m reaches 47.32 dBμA/m, and, if 0.16 pT is used as the threshold, the communication distance can reach 1446 m, which is significantly better than the traditional solution. This design marks the first instance of achieving kilowatt-class VLF effective radiation in a compact 51 cm-diameter magnetic loop antenna, offering a highly efficient solution for applications such as mine communication and geological exploration.

## 1. Introduction

Very-low-frequency (VLF, frequency range 3–30 kHz) radio waves play a crucial role in long-distance underwater navigation and communication due to their stable propagation characteristics and low attenuation rates [1,2,3,4]. However, traditional VLF transmitter antennas [5] (e.g., T-shaped antennas, umbrella antennas, etc.) are limited in their applications because of the wavelength limitation, large physical dimensions (which usually reach several square kilometers), and the need to lay a large-area ground network. Therefore, the construction of miniaturized VLF antennas has become a current research hotspot. Research and experiments have been conducted for some feasible solutions to promote the miniaturization of VLF antennas, such as mechanical antennas and magnetic resonant coupled antennas. Ning et al. proposed a dual-driven magnetoelectric antenna based on mechanical antennas, which achieved an effective distance of 170 m for communication through a pair of magnetoelectric heterostructures by utilizing the electrostatic effect [6]. Yang et al. designed and developed a magneto-mechanical transmission antenna of a simple, compact structure with low-power-consumption magneto-mechanical transmission antenna for ELF underwater communication up to 210 m [7]. Kurs et al. experimentally verified the feasibility of high-magnetic-field radiation by a double coil in a magnetic resonant coupling state [8]. On this basis, Zeng et al. proposed a magnetic resonant coupled metamaterial (MRCM) antenna with a dimensional near-field, adjustable frequency, and direction, which can realize effective communication up to 180 m in an open field [9]. However, the communication distance achieved by the above antenna is insufficient for many practical application scenarios.

In addition to the above antennas, magnetic loop antennas have attracted much attention owing to their small size, compact closed structure, and low ground coupling characteristics, providing a novel solution for miniaturized VLF antennas. Nanocrystalline materials can achieve high flux concentrations and greater reductions in energy loss owing to their excellent electromagnetic properties. Therefore, they are highly suitable for use in high-flux electromagnetic devices and can be integrated into electromagnetic devices to significantly reduce core losses and improve efficiency. However, they are mainly utilized in fields such as those involving transformers and motors. For example, Wu et al. [10] produced an isolation transformer based on nanocrystalline alloy materials for the impedance matching of a ring antenna, which effectively improved the radiation intensity of the ring antenna. The ring diameters of the small and large two-ring antennas were configured according to the actual application scenarios, where, for the ring diameter of the 0.3 m ring antenna, transmission distance was approximately 140 m (0.45 pT), and, for the ring diameter of the 2 m ring antenna, the effective communication distance was up to 340 m (0.8 pT). However, both can only withstand a 200 W input power. When used for mine communications and other site-restricted scenarios, the ring diameter of 2 is too large, whereas, for the 0.3 m ring antenna, the 140 m communication distance is relatively limited.

This study presents a very-low-frequency (VLF) antenna designed with a core composed of a nanocrystalline alloy material, characterized by its high-power input capacity, minimal loss, and compact structure. The antenna is made of high-carrying Leeds wire wound in a nanocrystalline alloy core and realizes high-power bearing through a large magnetic ring balun transformer and LC resonant network, which effectively improves the radiation performance of the VLF antenna. In addition, a high-voltage isolation capacitor was connected in series between the balun transformer and power amplifier to ensure a high power input to the antenna while blocking the DC bias and low-frequency noise. We analyzed the radiation enhancement mechanism of the proposed antenna using an equivalent circuit model and electromagnetic simulation. The field strength of the antenna at 120 m and 7.8 kHz was measured in an outdoor environment, which was up to 47.32 dBμA/m. The magnetic flux density is increased by 1800 times compared with that of the traditional loop antenna, and the maximum communication distance of the antenna can be expected to be up to 240 m If 0.16 pT is used as the communication threshold, the maximum communication distance of the antenna is expected to be up to 1446 m, which provides a potential solution for miniaturized low-frequency communication scenarios such as mine communication and geological exploration with reliable performance.

## 2. Basic Theory of Magnetic Loop Antenna

### 2.1. Radiation Performance Analysis

**Figure 1:**
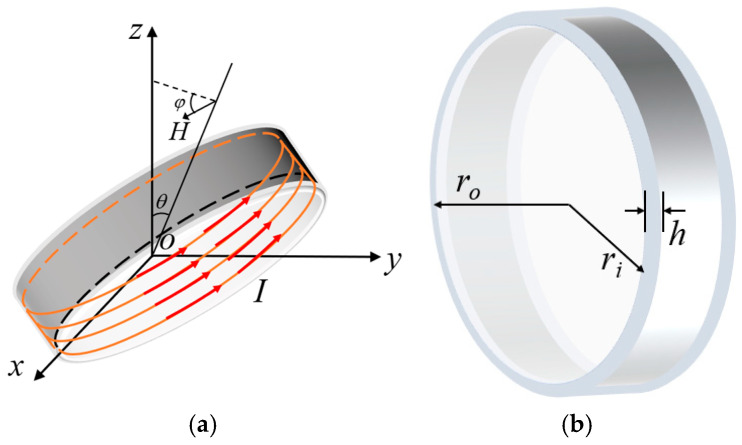
(**a**) Theoretical model of ring antenna, (**b**) toroidal core model.

Figure 1a,b shows the theoretical model and simulated impedance of a typical multi-turn coiled magnetic loop antenna, respectively. The expression for the near-field radiated field of an N-turn coiled loop antenna can be derived from Maxwell’s equations as

(1) $$H_{r}\approx \frac{n\mu_{r}IA}{2\pi r^{3}}\cos\theta \;H_{\theta }\approx -\frac{n\mu_{r}IA}{4\pi r^{3}}\sin\theta$$

where *H_r_* denotes the radial magnetic field component, *H_θ_* the tangential magnetic field component, *n* the number of turns of the coil, *μ_r_* the relative permeability of the core, *I* the output current, *A* the cross-sectional area of the magnetic ring, *r* the distance from the observation point to the field source, and *θ* the pole angle (angle from the coil axis).

Magnetic permeability reflects the response of a medium to a magnetic field. For an ideal magnetic circuit of infinite length and complete closure, the internal magnetic field of the magnetized material is *B* = *μ*_0_*μ_r_H*. For actual cores, constrained by finite dimensions and geometries, an effective permeability factor *m* exists. The magnetic permeability of a magnetized material is the value of its internal magnetic field of the magnetized material.

For a toroidal nanocrystalline alloy core, the magnetic induction can be expressed by the following equation:

(2) $$B=\frac{nIm\mu_{r}}{l_{m}}\;l_{m}=\pi (r_{i}+r_{o})$$

*l_m_* is the average magnetic circuit length of the toroidal magnetism; *r_i_* and *r_o_* are the inner and outer diameters of the core, respectively. At the desired operating frequency, a high radiated field strength can be achieved by selecting a suitable number of turns and diameter ratio. We have analyzed the effect of coil turns and core geometry on the radiation performance of the magnetic loop antenna using numerical analysis and a method of moments pair. The output current in Equation (2) can be changed by solving the output current using Ohm’s law under the input voltage *V*.

(3) $$B=nVm\mu_{r}/({R_{tot}}^{2}+{X_{L}}^{2})l_{m}\;R_{tot}=R_{dc}+R_{c}$$

where *R_tot_* is the loss resistance, *X_L_* is the reactance, *R_dc_* is the DC resistance, and *R_c_* is the core eddy current loss.

(4) $$\left\{\begin{array}{l} X_{L}=2\pi \mathrm{fL}\\ L=m\mu_{r}\mu_{0}N^{2}A/l_{m}\\ R_{c}=2\pi f\mu_{o}m\mu_{r}\tan\delta N^{2}A/\pi r_{o}\\ R_{dc}=\rho_{l}N\pi r_{o}/S \end{array}\right.$$

*ρ_l_* is the Leeds wire resistivity, *S* is the Leeds wire cross-sectional area, and tanδ denotes the core loss angle tangent. Bringing Equations (3) and (4) into (2) yields

(5) $$B=\frac{m\mu_{r}V}{2\pi s\sqrt{s^{2}f^{2}m^{2}{\mu_{r}}^{2}n^{2}A^{2}+(r_{o}+r_{i}{)}^{2}\rho^{2}{r_{o}}^{2}}}$$

In order to further investigate the effect of core geometry on the magnetic field strength of the magnetic loop antenna, the distribution of magnetic induction strength within 200 m of the magnetic loop antenna at a typical frequency (3 kHz) with different outer diameters *r_o_* and different thickness-to-diameter ratio $t=h/r_{o}=\left(r_{o}-r_{i}\right)/r_{o}$ is analyzed by Feko electromagnetic simulation software based on the method of moments. In the simulation setup, the input voltage is set to 60 V, the diameter of the excitation line is 6 mm, the conductivity is taken as 1.68 × 10^6^, the relative permeability of nanocrystalline alloy is taken as 5 × 10^4^, the conductivity is taken as 1 × 10^7^/18, and the X axis direction is the circumferential direction of the magnetic ring.

**Figure 2:**
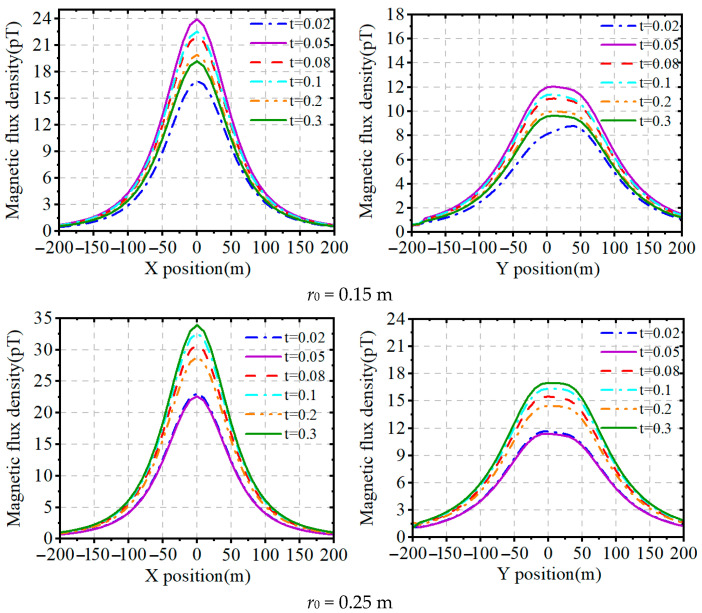

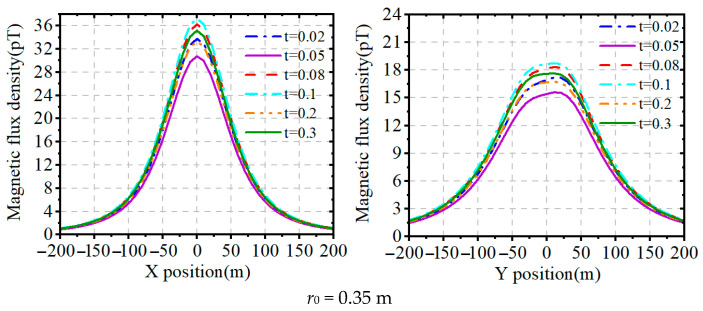
Magnetic induction distribution of magnetic loop antenna with different thickness-to-diameter ratios.

From Figure 2, it can be seen that, under the same thickness-to-diameter ratio, increasing the outer diameter can enhance the magnetic field strength, the circumferential magnetic field increases significantly, and the longitudinal magnetic field increases slightly or is flat. For medium ring radius (*r*_0_ = 0.25~0.35 m), the magnetic field strength is affected by the thickness-to-diameter ratio, and the overall trend increases with the increase in thickness-to-diameter ratio; for small ring radius (*r*_0_ ≤ 0.15 m), the magnetic field strength is determined by the ring diameter and thickness-to-diameter ratio. According to the antenna cost and different application scenarios, a different ring diameter and thickness-to-diameter ratio can be selected.

The thickness-to-diameter ratio affects the effective permeability factor *m*, which affects the magnetic induction strength of the magnetic loop antenna. From the data in Figure 2, we know that the magnetic field strength of the medium-ring antenna is proportional to the thickness-to-diameter ratio. In order to analyze the relationship between the effective permeability factor and the thickness-to-diameter ratio, we used Matlab 2024a numerical software to call the data from Figure 2 and then to analyze the data, as shown in Table 1.

**Figure 3:**
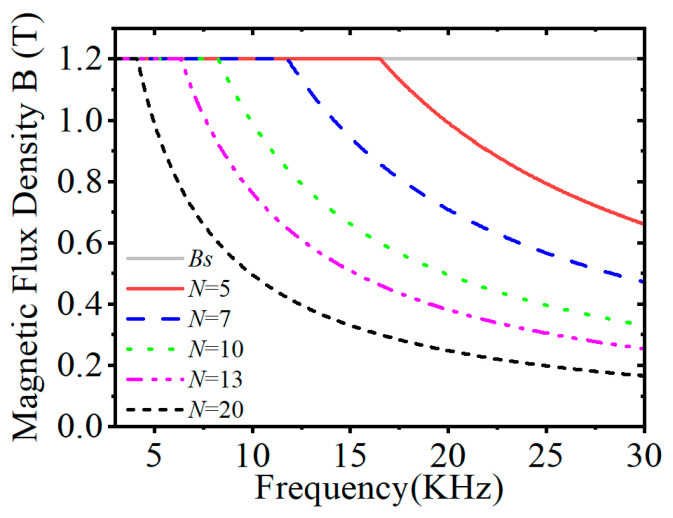
Magnetic induction for different coils.

In addition, we also analyze the effect of different coil turns on the magnetic induction intensity when the ring diameter is certain, as can be seen in Figure 3. Due to the existence of nanocrystalline alloys saturated magnetic flux, there are different cutoff frequencies for different coil turns: the more coil turns, the lower the cutoff frequency and the more gentle the change in magnetic induction intensity with the frequency. However, fewer coil turns in the high-frequency band still does not produce low magnetic induction intensity. Therefore, we can choose the appropriate number of turns according to the different operating frequencies and application scenarios.

### 2.2. Nanocrystalline Core Ring Antenna

#### 2.2.1. Nanocrystalline Material Properties

From Equation (5), it can be seen that the magnetic induction strength of the magnetic loop antenna is directly proportional to the relative permeability of the core, and there are large differences in the performance of different cores due to their processing and material composition. The permeability, coercivity, saturation magnetization strength, and resistivity of different core materials are given in Table 2. From Table 2, it can be found that silicon steel has a high magnetic flux density, although the high coercivity leads to high hysteresis loss, limiting its potential application in VLF band scenarios. Ferrite has low saturation magnetic density and high hysteresis loss, which is not suitable for high-flux-density scenarios such as magnetic loop antennas. Amorphous alloys are excellent in permeability and coercivity, and their high-temperature stability and losses are slightly lower than those of nanocrystalline alloys. Nanocrystalline materials have low coercivity, high permeability, high saturation magnetic induction, and high temperature stability for efficient magnetization and effective permeability processes, which can be effectively used for VLF communications.

**Figure 4:**
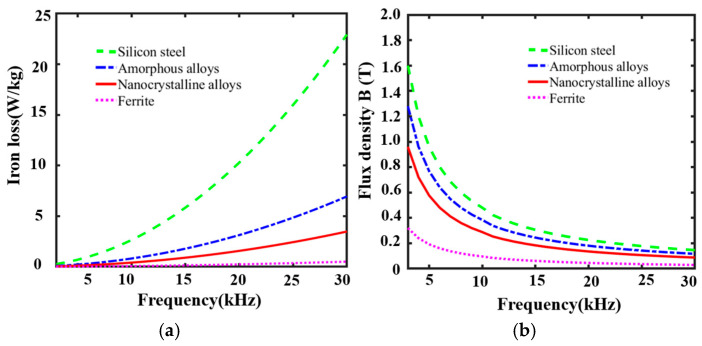
(**a**) Iron loss of different core materials; (**b**) Magnetic flux of different core materials.

As can be seen from Figure 4, the iron loss of different materials increases with the rise in frequency. The micro-crystalline alloy not only has lower iron loss in the frequency range of 3~30 kHz, but also maintains a high magnetic flux density. Its magnetic flux is slightly lower than that of silicon steel, but significantly higher than that of ferrite. And the effective magnetic flux of about 0.6 T is still maintained at 7.8 kHz, which indicates that the nanocrystalline alloys can still maintain high magnetic properties at high frequencies, with strong demagnetization resistance and broadband stability.

**Figure 5:**
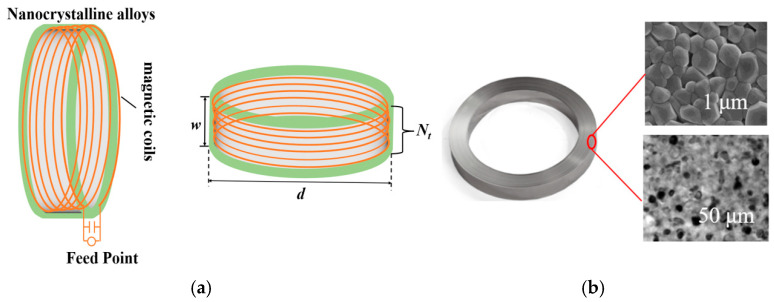
(**a**) Geometrical model of a multi-turn coil loop antenna. (**b**) Nanocrystalline alloy.

The nanocrystalline alloy effectively suppresses the high-frequency eddy current loss through grain refinement and disordered structure design (as shown in Figure 5b), which results in excellent performance in the frequency range of more than 3~20 kHz. And the low coercivity, high permeability, and high-saturation magnetic induction strength make it suitable for high-flux-density application scenarios.

#### 2.2.2. Ring Antenna Design Based on Nanocrystalline Materials

We have designed a magnetic loop antenna with 10 turns of coil, 0.51 m ring diameter, and 0.08 thickness-to-diameter ratio. Figure 3 shows the geometry of the proposed antenna, which consists of two parts: a ring-shaped nanocrystalline alloy core (Fe-Si-B-Nb-Cu) and a Leeds wire (wire diameter 6 mm); the detailed parameters of the antenna and the nanogold alloy material are shown in Table 3.

### 2.3. Matching Design

**Figure 6:**
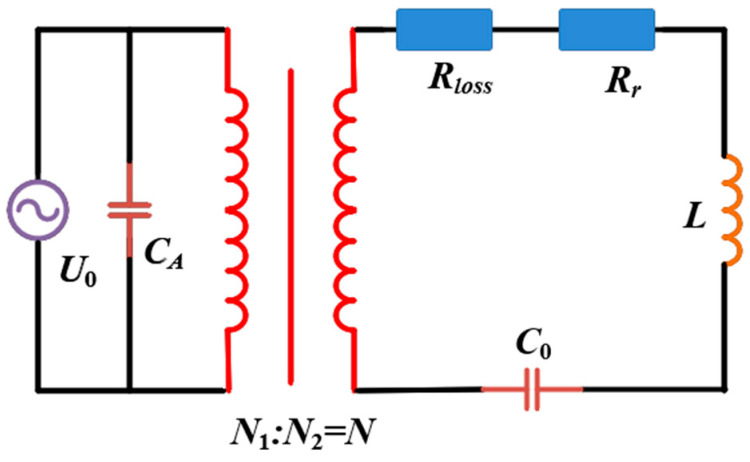
Equivalent circuit diagram of the ring antenna.

According to Equation (5), it can be seen that the radius and the number of turns of the ring cannot be increased infinitely due to the limitation of volume and weight; therefore, increasing the transmit current is an effective means to enhance the magnetic induction strength of the magnetic ring antenna. In order to maximize the current transmission, an equivalent circuit is constructed to demonstrate the radiation enhancement performance of the proposed high-power VLF magnetic ring antenna based on an LC resonant network with a high-power balun transformer, as shown in Figure 6. In the figure, *R_r_* is the radiation resistance and *C_A_* is the isolation capacitor used to block DC bias and low-frequency noise. The equivalent variable inductance of the magnetic loop antenna can be obtained from Equation (4).

**Figure 7:**
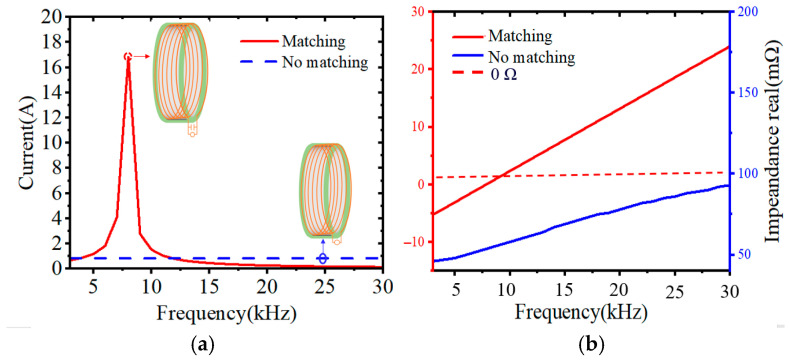
(**a**) Resonant response at 7.8 kHz. (**b**) Impedance characteristics at 7.8 kHz.

In order to increase the output current, a resonant capacitor _C0_ is inserted in series so that the magnetic loop antenna resonates in series to obtain a high current output; the value of the series capacitance can be calculated from $\sqrt{LC_{0}}=1/2\pi f$. The value of the series capacitance can be calculated; Figure 7a gives the resonant response of the loop antenna at 7.8 kHz frequency when the input voltage is 60 V and the input current is 0.86 A. It can be found that the antenna current appears to have an obvious peak of 16.8 A around 7.8 kHz, which is amplified by about 20 times compared with the input current. This implies that the LC resonant network based on the magnetic coupling resonance characteristic can greatly improve the radiation performance of the antenna.

When solving the ring antenna reactance, because the nanocrystalline alloy core is wound layer by layer, there is an air gap between adjacent layers. Its relative effective magnetic flux rate cannot be taken as 50,000; it is more appropriate to take 11,000 after the correction of the subsequent test data and after checking the effective permeability of the ring magnetic core under different diameter ratios in Table 1. Table 4 shows the comparison between the simulation results and theoretical calculations in the tuned state. The two impedance calculations are more consistent, the real part is basically the same, while the simulation of the imaginary part is large because of the consideration of the turn-to-turn capacitance between the neighboring coils, taking into account the nonlinear change in the magnetic core permeability with frequency. However, the core permeability is regarded as a fixed value in the theoretical calculation, and the value is taken as 11,000.

To further increase the output current, the loss resistance needs to be reduced. The loss resistance of the antenna is mainly composed of the eddy current loss of the core and the DC resistance of the Leeds line.

From Equation (4), it can be seen that increasing the effective permeability of the magnetic ring antenna can reduce the eddy current loss of the magnetic core. And *μ_eff_* is related to the inductance of the ring antenna itself. In the case of a certain core, different winding methods and numbers of turns of the coil will affect the antenna inductance. Figure 7 gives the inductance as well as the magnetic induction strength of the loop antenna with different winding methods under 60 V output voltage.

**Figure 8:**
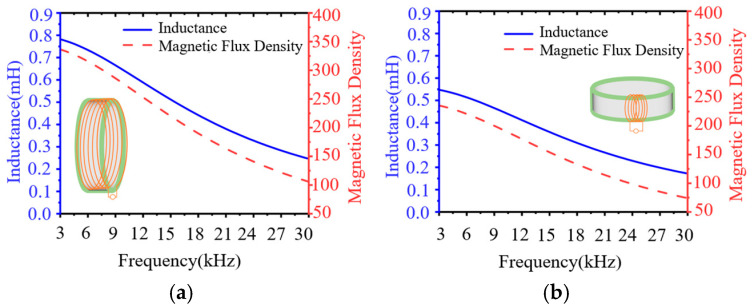
(**a**) Circumferential winding. (**b**) Radial winding.

From Figure 8a,b, it can be found that the inductance and flux rate density of both winding methods show a gradual decrease with increasing frequency. The toroidally wound coil exhibits higher inductance in the frequency range of 3 kHz to 30 kHz of 0.8 mH at 3 kHz, with a smoother inductance decay rate. Radial winding results in a 66–69% decrease in inductance due to magnetic leakage and a 1.5-fold increase in magnetic induction. This is due to the fact that the effective cross-sectional area of the magnetic circuit is increased because the Leeds wires are evenly distributed along the circumference of the core in the circumferential direction of the winding. The spacing of adjacent coils is consistent, which reduces the skin effect and proximity effect between the coils and improves the effective permeability of the core, thus obtaining a higher inductance value. For the choice of the number of turns of the coil, analyzing the magnetic induction intensity of different coils in Figure 3, it can be seen that, the greater the number of turns of the coil, the lower the frequency of the core to reach saturation flux. For the electromagnetic wave, the lower the frequency, the stronger the ability to penetrate the medium, making it more suitable for mine communication, geological exploration, and other scenarios. However, the excessive number of turns brings the problem of weight increase and the problem of difficulty in matching the reactance. Comparing the data in Figure 3 and Table 4, it can be seen that the cutoff frequency of the 10-turn coil is around 7.8 kHz, and the tuning capacitance is only 2.3 μF, so we chose 10 turns for the number of coil turns.

High output current usually means high power capacity. In order to improve the power withstanding of the magnetic core, the ring antenna core is made of nanocrystalline alloy material, and a magnetic ring with an outer diameter of 51 cm, an inner diameter of 49 cm, and a thickness of 2 cm is fabricated. Ten turns are wound circumferentially using a Leeds wire (with a current carrying capacity of 40 A) that utilizes a wire diameter of 6 mm. A value of 7.8 kHz is selected as the resonant frequency, and, from the simulation results in Figure 7a, it can be seen that, under the condition of 60 V output voltage, the output current of the ring antenna is 16.8 A. At this time, the output power is 1008 W.

**Figure 9:**
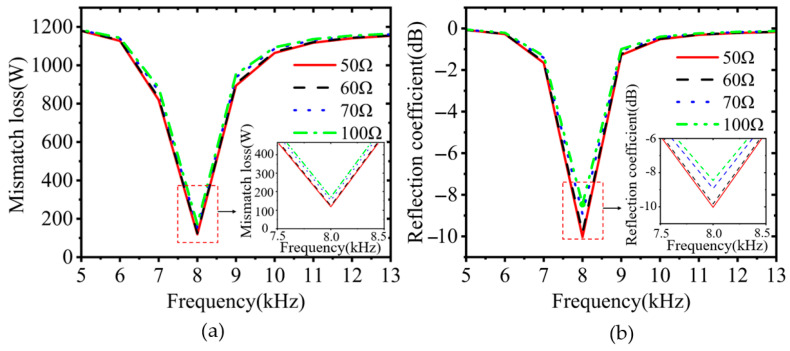
Simulation of (**a**) reflection coefficient and (**b**) mismatch loss power.

The actual size of the VLF magnetic loop antenna is much smaller than the electrical length of its corresponding frequency, so the real part of the antenna is very small—only 10^−1^ Ω. The RF output port of the power amplifier is 50 Ω. If the signal is transmitted directly through the 50 Ω coaxial cable without impedance matching between the two, this will lead to impedance mismatch and increased reverse power. Under high power input, this can even damage the instrument. Therefore, it is necessary to use the Barron impedance converter for impedance matching to amplify the real part of the antenna to 50 Ω by a factor of N2, where N is the turns ratio of the primary coil to the secondary coil. According to the simulation results in Figure 9, the closer the antenna output impedance is to 50 Ω, the smaller the reflection coefficient and the lower the mismatch power loss the magnetic loop antenna exhibits under the conditions of fixed input power (1200 W) and consistent resonant frequency (7.8 kHz).

In addition, to ensure that the Barron impedance converter can withstand 1200 W input power, an impedance converter with an outer diameter of 30 cm, an inner diameter of 25 cm, and a thickness of 3 cm is fabricated using ferrite material. And the power capacity of the transformer made with a copper conductor of 6 mm wire diameter can be expressed by the following equation [11]:

(6) $$P_{\max}=KB_{s}\mathrm{fA}A_{w}J\;A_{w}=(r_{o}-r_{i})\cdot H$$

where *P_max_* is the maximum power capacity, *K* is the topological constant related to the core geometry and winding method, *A_w_* is the core window area, *J* is the wire current density, and *H* is the core thickness. For the circumferential winding of the ring core, *K* value is generally 0.3~0.5; using Formula (8) for calculations, we take the *K* value of 0.3 to obtain the maximum power of the ring in order to withstand the power of 3370 W.

## 3. Experimental Verification

**Figure 10:**
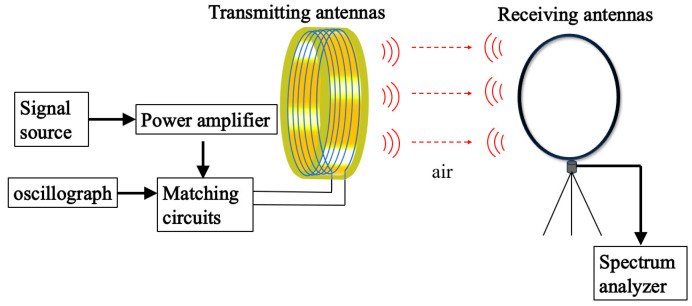
Schematic of VLF ring antenna magnetic field strength test.

In order to evaluate the transmit performance of the ring antenna, this test was conducted with a homemade VLF ring antenna with a ring diameter of 0.5 m as the subject, and the near-field test based on the magnetic ring antenna was carried out, as shown in Figure 10.

In Figure 10, the VLF signaling system consists of a signal source (HDG2032B, Qingdao Hantai, Qingdao, China) connected in series to a power amplifier (DH17834A, Beijing Dahua, Beijing, China), which then outputs a 3.6 V excitation signal through a matched capacitor to drive a transmitter loop antenna with an operating frequency of 7.8 kHz. Before the signal source outputs the signal, the reactance value of this antenna at 7.8 kHz is measured to be 0.88 + j7.48 by using an RLC meter (VICICR4091A, Uli De, Shenzhen, China), and the actual measured resistance value is 0.4 ohms larger than the simulated value, which may be due to the machining error in the actual process. The required tuning capacitance of the loop antenna at 7.8 kHz output is found to be 3 μF by the LC series resonance theorem. The matched capacitor was connected to an oscilloscope (MSOX3024T, KEYSIGHT, Xi’an, China) for monitoring the output current of the transmitting loop antenna, and the receiving loop antenna (HFH2-Z2E, ROHDE&SCHWARZ, Xi’an, China) was set up 120 m away from the transmitting loop antenna; the field strength level values were recorded using a spectrometer (ROHDE&SCHWARZ, ROHDE&SCHWARZ, Xi’an, China).

**Figure 11:**
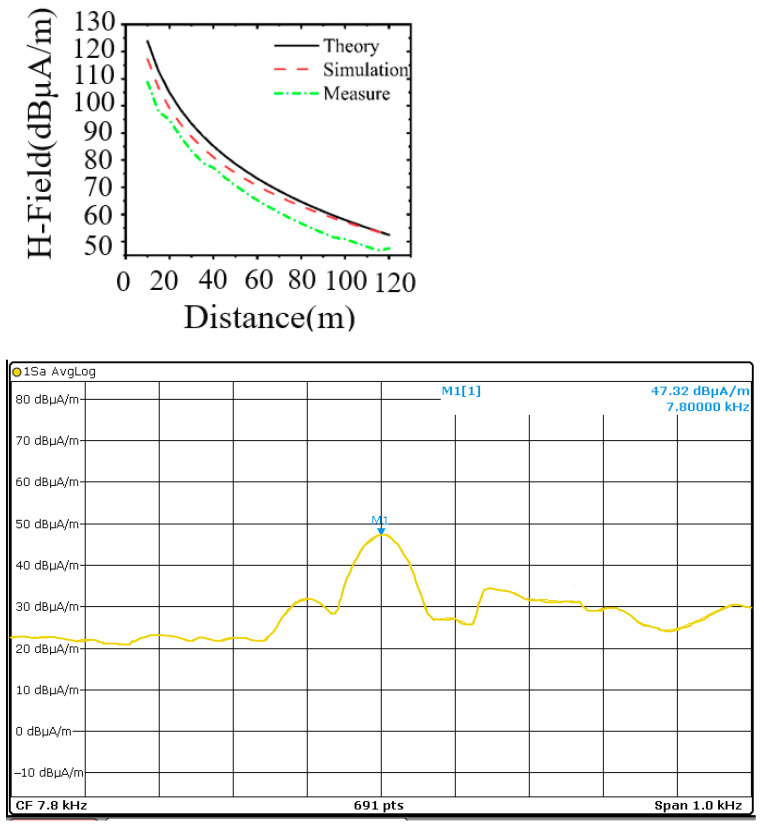
Magnetic field strength at 120 m for VLF loop antenna with 7.8 kHz frequency.

**Figure 12:**
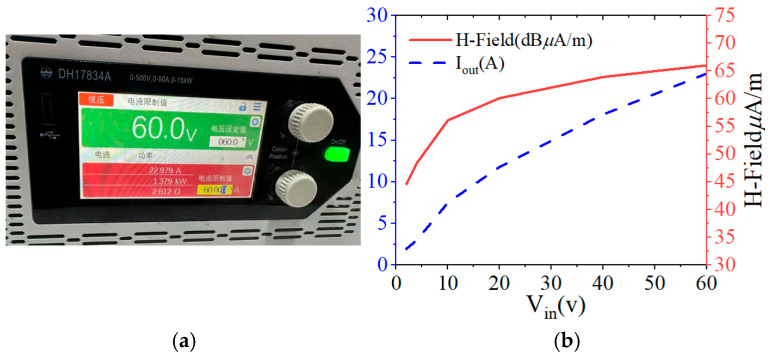
VLF ring antenna magnetic field strength test. (**a**) The voltage and current of the antenna during actual measurement. (**b**) The measured curves of current and magnetic field strength varying with frequency.

The test process utilizes the power amplifier to set different output voltages and rotate the orientation of the receiving loop antenna to observe the field strength value on the spectrometer until the field strength value reaches the maximum value to stop rotating and record the VLF transmitter antenna at 70 m. The induced field strength of the VLF antenna at 70 m is recorded. The magnetic field strength of the magnetic loop antenna at 70 m is shown in Figure 11. The magnetic field strength of the magnetic loop antenna at 70 m under different output voltages is given in Figure 12, The test environment is relatively noisy, and the ambient noise floor is 19 dBμA/m. From Figure 12a, it can be seen that the antenna is at an output frequency of 7.8 kHz, and the input voltage is 60 V, at which time the output current is 23 A and the output power reaches 1380 W. The field strength value at 70 m still has 66 dBμA/m, with strong sensing ability.

In order to evaluate the antenna sensing capability and to determine the maximum attenuation distance of its field strength as it propagates in air, the values of the induced field strength of the receiving loop antenna relative to the magnetic loop antenna were measured at different locations under 1200 W output power. Due to the site constraints, we only carried out the test at a range of 120 m. A comparison of the theoretical, simulated, and measured results of the induced field strength over distance in the range of 10 to 120 m when the VLF loop antenna operates at 7.8 kHz is shown in Figure 11. The theoretically calculated and simulated values are basically the same, but they are slightly higher than the simulated values in the low-frequency band because the simulation calculations take into account the ground loss. The measured values are about 4–5 dBμA/m smaller compared with the simulated data, which is attributed to the nonlinear change in the core impedance at high voltage not considered in the theoretical model. The VLF electromagnetic wave generated by this antenna decays rapidly in the near field, and the magnetic field strength of the loop antenna at 120 m is 47.32 dBμA/m. It is also known from Equation (1) that the magnetic field strength decays proportionally to the third power of the distance. If 0.16pT is used as the threshold, the calculated communication distance can reach 1446 m, thus effectively realizing the miniaturized VLF transmitter antenna.

A comparison of the proposed magnetic loop antenna with other existing reported advanced and miniaturized loop antennas is shown in Table 5. Most of the miniaturized loop antennas reported so far are based on the electrostrictive effect of piezoelectric materials or the physical motion (translation, rotation) of permanent magnet materials to realize the miniaturization of the antenna. It has been reported that the communication distance of a DDME antenna [6] can reach 170 m (1.2 pT), which is already a high level. However, the limited input power is the main factor limiting the further improvement of the radiation capability of this type of antenna. In contrast, the nanocrystalline material antenna proposed in this paper achieves significant long-distance communication over 120 m at 7.8 kHz. The radiation intensity of this antenna at a distance of 120 m is 47.32 dBμA/m, which is at the higher level of currently reported miniaturized loop antennas.

## 4. Summary

In this paper, a VLF magnetic loop antenna consisting of a multi-turn coil and a toroidal nanocrystalline alloy core structure with high power input, low loss, directionally adjustable characteristics, and compact structure is proposed. A quantitative correlation model between the core thickness-to-diameter ratio (t = 0.02) and the effective permeability factor (m = 0.22) is established for the first time, and the effective permeability is increased to 11,000. The neighbor effect is suppressed by using the toroidal winding process, which improves the magnetic induction strength by 1.5 times and reduces the loss, laying the foundation of high-frequency and high-power radiation. The antenna with 7.8 kHz as the working frequency is fabricated, and, under the condition of 1200 W input power, the measured magnetic field strength at 120 m reaches 47.32 dBμA/m, which is consistent with the theoretical and simulation results and proves its strong radiating capability.

Compared with the traditional ring antenna, power capacity increased by 6 times, and the magnetic flux density improvement is significant, increasing by 1800 times. The theoretical model predicts that the antenna’s maximum communication distance can be up to 1446 m (with 0.16 pT as the threshold), which provides a portable solution for the scenarios of mine tunneling and geological exploration. The cascaded LC resonance and balun transformer architecture provides a universal technology path for VLF magnetic loop antennas. It further expands the application scenarios of VLF miniaturized antennas.

## Figures and Tables

**Table 1:** Effective permeability factors of magnetic rings with different thickness-to-diameter ratios.

Thickness-to-Diameter Ratio	Effective Permeability Factor *m*	Effective Permeability *μ_eff_*
0.02	0.1654	8270
0.05	0.16208	8104
0.08	0.22	11,000
0.1	0.2332	11,660
0.12	0.33218	16,609
0.15	0.29936	14,968
0.18	0.29856	14,928
0.2	0.20508	10,254
0.23	0.30824	15,412
0.25	0.34852	17,291
0.28	0.33508	16,754
0.3	0.37804	18,902

**Table 2:** Material properties of different core materials.

Materia	Permeability	*B_s_*	Coercion	Resistivity	*T_c_*
silicon steel	2000~5000	1.5~2.0 T	10~50 A/m	4 × 10^−5^ Ω·m	730 °C
amorphous alloy	20,000~30,000	1.2~1.6 T	1~10 A/m	1 × 10^−6^ Ω·m	415 °C
nanocrystalline alloys	30,000~50,000	1.0~1.4 T	0.5~5 A/m	1 × 10^−7^ Ω·m	560 °C
ferrite	2000~10,000	0.3~0.5 T	100~500 A/m	1 × 10^−3^ Ω·m	180 °C

**Table 3:** Detailed parameters of a multi-turn coil loop antenna.

Sign	Clarification	Value
**Ring antenna (with core)**		
*w*	Antenna width	50 mm
*d*	Antenna ring diameter	510 mm
*w_t_*	Antenna weight	2.5 kg
*N_t_*	Number of turns	10
*a*	Leeds wire diameter	6 mm
**Nanocrystalline Alloy Cores**		
*μ_r_*	Relative permeability	50,000
*ρ_m_*	density	7.2 g/cm^3^
*cρ*	Resistivity	1.8 μΩ/m
*B_s_*	Saturation magnetic field density	1.2 T
tan*δ*	Loss angle tangent (math.)	0.01

**Table 4:** Theoretical and simulated values of ring antenna reactance values.

Methodologies	Frequency	Turns	Ring Diameter	Leeds Wire Diameter	Ring Impedance	Tuning Capacitor
Theory	3 kHz	10	0.51 m	6 mm	0.16 + j16.58Ω	3.2 μF
	7.8 kHz	10	0.51 m	6 mm	0.44 + j43.12Ω	0.47 μF
	13 kHz	10	0.51 m	6 mm	0.71 + j71.88Ω	0.17 μF
	18 kHz	10	0.51 m	6 mm	0.99 + j99.52Ω	0.08 μF
	23 kHz	10	0.51 m	6 mm	1.27 + j127.17Ω	0.05 μF
	28 kHz	10	0.51 m	6 mm	1.54 + j154.81Ω	0.04 μF
Simulation	3 kHz	10	0.51 m	6 mm	0.16 + j3.3Ω	16 μF
	7.8 kHz	10	0.51 m	6 mm	0.48 + j8.7Ω	2.3 μF
	13 kHz	10	0.51 m	6 mm	0.75 + j14.1Ω	0.9 μF
	18 kHz	10	0.51 m	6 mm	1.1 + j19.5Ω	0.45 μF
	23 kHz	10	0.51 m	6 mm	1.4 + j24.9Ω	0.27 μF
	28 kHz	10	0.51 m	6 mm	1.7 + j30.3Ω	0.18 μF

**Table 5:** Performance comparison of different antennas.

Type of Program	Sizes	Freq	Power	Magnetic Flux/ Transmission Distance	References
Magnet-based Mechanical antenna	-	1.6 kHz	20 W	5 m (50 pT)	[12]
Loop antenna	30 cm	15 kHz	200 W	135 m	[10]
Relay waveguide system	22 cm	125 kHz	-	40 m	[13]
Relay waveguide system	12 cm	1 kHz	20 W	25 m	[14]
Metamaterial antenna	18 cm	41.4 kHz	200 W	180 m (0.16 pT)	[9]
Nanomaterials antenna	51 cm	7.8 kHz	1200 W	1446 m (0.16 pT)	This work

## Data Availability

Data sharing is not applicable. No new data were created or analyzed in this study.
